# Hempseed By-Product in Diets of Italian Simmental Cull Dairy Cows and Its Effects on Animal Performance and Meat Quality

**DOI:** 10.3390/ani12081014

**Published:** 2022-04-13

**Authors:** Castro Ndong Ncogo Nchama, Carla Fabro, Mario Baldini, Elena Saccà, Vinicius Foletto, Edi Piasentier, Angela Sepulcri, Mirco Corazzin

**Affiliations:** Department of Agricultural, Food, Environmental and Animal Sciences, University of Udine, 33100 Udine, Italy; ndongncogo.castro@spes.uniud.it (C.N.N.N.); carla.fabro@uniud.it (C.F.); mario.baldini@uniud.it (M.B.); elena.sacca@uniud.it (E.S.); vinicius.foletto@uniud.it (V.F.); edi.piasentier@uniud.it (E.P.); angela.sepulcri@uniud.it (A.S.)

**Keywords:** hempseed cake, *Cannabis sativa* L., cull cows, fat acids profile, carcass traits

## Abstract

**Simple Summary:**

At the end of their productive career, dairy cows are culled without a fattening period and, therefore, sold at a low market price. On the other hand, the cultivation of hemp (*Cannabis sativa* L.) is increasing, and hempseed cake is a by-product obtained during the mechanical extraction of the seed. Hempseed cake is rich in polyunsaturated fatty acids, so its use in the diets of cull cows could probably improve meat value and contribute to pursuing the objectives of a circular economy. In this study, hempseed cake was added to the diets of culls cows for the first time with the aim to assess its effect on animal performance and meat quality. The results of the present paper suggest that hempseed cake did not affect animal performance and the characteristics or the fatty acids profile of the meat. From this point of view, although no improvement in the level of unsaturated fatty acids in meat was found, this study showed that hempseed cake can replace soybean meal, thus improving the circular economy.

**Abstract:**

Cull dairy cows are important contributors to total beef production in the USA and in Europe. Hempseed cake is a by-product of oil production and it is rich in unsaturated fatty acids (FA). This study aimed to investigate the effect of adding hempseed cake to the diet of Italian Simmental (IS) cull dairy cows on performances and meat quality. Twenty-six cull dairy cows were divided into three dietary groups: hay-based, corn silage-based and pasture-based diets. Within each group, the animals were equally divided into two treatments according to the protein source of the concentrate: hempseed cake (HEMP) or soybeans meal (SB). The trial lasted four months. HEMP showed similar in vivo performance and carcass characteristics, such as average daily gain (*p* > 0.05) and dressing percentage (*p* > 0.05), compared with SB. Meat characteristics, such as ether extract content and Warner–Bratzler shear force, were also similar between experimental groups (*p* > 0.05). Considering FA composition, HEMP showed similar saturated FA and polyunsaturated FA content (*p* > 0.05) but lower desirable fatty acids (*p* < 0.05) content and a tendentially lower hypocholesterolemic/hypercholesterolemic ratio (*p* < 0.10) than SFA. Hempseed cake can substitute soybean in the diet of cull dairy cows without effects on performance or meat quality.

## 1. Introduction

Cull cows contribute substantially to beef production. Indeed, about 30% of the total beef produced in 2018 in Europe came from cows [1], and in the same year, 10% of total beef production in the USA derived from cull dairy cows [2]. These figures could be explained by the herd culling rate ranging from 23% to 36% [3], which has been increasing since 2004 [2]. Smith et al. [4] reported that low production, udder disease or lesion and reproduction problems are the main causes of culling. Due to low commercial values [5], farmers often slaughter cull dairy cows immediately after removal from the herd without a previous fattening period [6]. The Italian Simmental (IS) is a dual-purpose (meat and milk) breed belonging to the Simmental population, which, with more than 40 million animals present on all continents, is one of the most important cattle populations in the world. Animals belonging to this population have a greater capacity to produce meat and lean tissue than those of dairy breeds and, therefore, have the potential to provide increased income to the farmer if fattened [7]. On the other hand, improving the fatty acids (FA) profile, by increasing unsaturated FA and reducing the n-6/n-3 polyunsaturated FA (PUFA) ratio [8], may be a strategy to improve the acceptability of cull dairy cow meat relative to consumers and, thus, its commercial value. However, few studies are available on cull dairy cows as a beef source [2].

Hemp (*Cannabis sativa* L.) is considered as a sustainable bioenergetic crop that can be used in the production of bioethanol, biogas and combustion biomass [9] and can be cultivated in large parts of the world. Moreover, the reproductive and vegetative organs are rich in secondary metabolites, such as cannabinoids, terpenoids and flavonoids [10], which, once extracted, have various applications in drugs, cosmetics, bio-pesticides and antimicrobials [11]. The cultivation of hemp is expected to increase in the next few years [11], and the area of hempseed in Europe increased by more than 600% from 1993 to 2018 [12]. Hemp by-products, although underutilized, could be used as novel feed for ruminants [13]; among these by-products, hempseed cake, which is obtained together with oil during the mechanical extraction of the seed, appears to be of particular interest for animal feeding. It has a high biological value of protein with an amino acid profile comparable to that of soybean [14]. The fat content is around 10% of the dry matter (DM) with an FA profile particularly beneficial to humans. In fact, about 70% of total FAs are linoleic (C18:2n-6) and linolenic (C18:3n-3) acid, which are essential FAs because the human body is unable to synthetize them. Moreover, hemp has a C18:2n-6/C18:3n-3 ratio around 2:1–3:1, which is considered ideal for human health [14,15,16].

Very few studies are available on the use of hempseed in cattle diets. Available studies considered growing animals [17,18,19,20] and lactating cows [21]; to our knowledge, there is no research on cull cows. In a very recent review, Bailoni et al. [12] argued that more data are needed to determine the effects of dietary inclusion of hempseed cake on cattle.

Therefore, the aim of this study was to investigate the effects of adding hempseed cake to the diet of IS cull dairy cows on performances and meat quality.

## 2. Materials and Methods

### 2.1. Animals and Treatments

Twenty-six multiparous Italian Simmental cull dairy cows were divided into three dietary groups: hay-based (H, n = 10), corn silage-based (CS, n = 8) and pasture-based (Pa, n = 8) diets. Within each group, the animals were equally divided into two treatments based on the protein source of the concentrate: hempseed cake (HEMP) or soybeans meal (SB). The trial lasted four months after a one-month adaptation period. At the beginning of the trial, HEMP and SB groups had similar initial body weight (609.6 ± 13.87 kg), age (86.8 ± 6.60 months) and body condition score (3.14 ± 0.087 points) [22]. The animals that belonged to H and CS groups were loose-housed in one pen (6 × 19 m), each with sawdust bedding. During the experimental period, all cull cows in the Pa group were kept on the same pasture under rotational grazing, and twenty paddocks with a two-day rotation schedule were considered. The area of each paddock was 0.11 ha on average. Particular care was taken to ensure that pasture availability was much higher than the estimated DM intake of the animals [23]. The pasture was plain, located at an altitude of 186 m. a.s.l. and belonged to the class *Molinio-Arrhenatheretea*. In particular, the pasture comrpised grass (50%, mainly by *Festuca pratensis*, *Lolium perenne*, *Dactylis glomerata* and *Holcus lanatus*), legumes (15%, mainly by *Trifolium repens*, *Trifolium pratense* and *Lotus corniculatus*) and forbs (35%, mainly by *Tarassacum officinale*, *Plantago lanceolata*, *Centaurea nigrescens* and *Achillea millefolium*). The experimental grazing period was from June to September. The average temperature was 21.6 °C, the average precipitation was 5.7 mm/d and the number of days with at least 1 mm of rain was 49.

Cull cows belonging to H and CS groups received forage ad libitum and were individually supplemented with concentrates offered in twice daily meals. Pa fed only pasture and concentrate. In particular, in the Pa group, concentrate was offered through an automatic feeding system to which animals had individual access during the grazing period. Within dietary group H, 7.4 kg of a concentrated made up of barley (23%), wheat (45%), wheat bran (19%), soybean meal (7%), minerals and vitamins (6%) was offered to SB, and 7.7 kg of a concentrated made up of barley (22%), wheat (43%), wheat bran (18%), hempseed cake (11%), minerals and vitamins (6%) was offered to HEMP. Within dietary group CS, 3.2 kg of a concentrated made up of maize (43%), gluten meal (30%), soybean meal (15%), minerals and vitamins (12%) was offered to SB, and 4.1 kg of a concentrated made up of maize (46%), gluten meal (26%), hempseed cake (19%), minerals and vitamins (9%) was offered to HEMP. Within dietary group Pa, SB received 6.0 kg on average of a concentrated made up of barley (53%), wheat (16%), wheat bran (20%), soybean meal (7%), minerals and vitamins (5%), and HEMP received 7.6 kg on average of a concentrated made up of barley (52%), wheat (16%), wheat bran (19%), hempseed cake (9%), minerals and vitamins (4%). The composition and quantity of concentrates offered were in agreement with a target average daily weight gain of 1.1 kg/d for cull cows [23] and respected the maximum incorporation rate of hempseed cake in the diet of cull cows, 5%, as suggested by EFSA [24]. 

### 2.2. Measurements and Sample Collection

In order to assess the individual forage intake, animals in H and CS groups were equipped with a noseband pressure sensor (RumiWatch system, ITIN-HOCH GmbH, Liestal, Switzerland) and eating and rumination time, eating and rumination chews and number of boluses were recorded. The relationship between these variables and forage intake was studied during one week in the adaptation period [25]. For the Pa group, the animals’ grass intake was assessed by considering the difference between pre-grazing and post-grazing herbage mass and the regression equation of Vazquez and Smith [26]. The herbage mass was evaluated by a direct method [27]; specifically, herbage was harvest using a hand mower by the same researcher to reduce sampling errors in three quadrants (1 mq each) per paddock, dried and weighed. Forage and concentrate samples were collected every two days for the H and CS groups, as well for concentrate samples in the Pa group. The samples of herbage selected by each cull cows in Pa group were daily collected by a trained researcher in accordance with hand-plucking techniques [28] and bulked weekly.

During the experimental period, the cull cows were weighed individually every two weeks. The animals were slaughtered in an EU-licensed abattoir about 40 km far from the farm. The hot carcass weights were recorded, and the dressing percentages were calculated. During the slaughter procedure, the pH of the rumen fluid was measured using a pH-meter (Hanna, HI 8424, Padova, Italy) equipped with a glass electrode (Crison, 5232, Barcelona, Spain). Carcasses were graded for conformation and fatness (EU Reg. No 1208/81 and 1026/91) and kept at 4 °C. Forty-eight hours after slaughter, a sample of *longissimus thoracis* (LT) m. (6a–7a rib) was collected from the left side of the carcass of each animal and divided into two parts. On the first part, the pH was measured as for rumen fluid, and then the sample was vacuum freeze dried (LyoQuest-55, Azbil Telstar Technologies S.L.U, Terrassa, Spain) and stored at −20 °C until proximate and FA analyses. The second part was aged for 14 days at 4 °C, and the color, cooking loss and shear force were determined.

Color measurements were made, after a blooming time of 45 min, using a portable spectrophotometer (CM 2600d; Konica Minolta, Tokyo, Japan), with an 8 mm aperture, Standard Illuminant D65 light source and 10 viewing angle geometry. The values recorded, according to the standard conditions of the Commission Internationale de L’Eclairage, included lightness (L*), redness (a*) and yellowness (b*).

Slices of LT muscle of 2 cm thickness were cooked in plastic bags, using a water bath, until they reached 75 °C at the core [29]. The internal temperature was monitored by a thermocouple (Thermocouple Thermometer HD 9016; Delta Ohm, Padua, Italy). Each slice of meat was weighed before and after cooking. The cooking loss was calculated as the difference between the weight before and the weight after cooking and expressed as a percentage of the initial weight.

The Warner–Bratzler shear force (WBSF) was determined on cooked meat after cooking-loss analysis. At least 7 replicated cylindrical cores (15 mm diameter) were sheared, perpendicularly to the muscle fibers’ direction, using a Warner–Bratzler device with a triangular opening blade (60°) in the shear blade (1 mm thickness) mounted on a texture analyzer (Lloyd TA Plus; Lloyd, Borgnor Regis, UK) and operating at a crosshead speed of 100 mm/min. 

### 2.3. Chemical Analysis

The forage and concentrate samples were dried in a forced draft oven at 60 °C for 48 h. After the drying process, the samples were ground to a homogeneous mass in a mill with a 1.0 mm grid and then used for crude protein (CP), crude fiber, ether extract and ash analyses in according to the Association of Official Analytical Collaboration (AOAC) [30]. The net energy for lactation (NEl) was calculated according to National Institute for Agricultural Research (INRA) standards [23]. The proximate analysis of beef samples was also performed according to AOAC [30].

Lipid extraction was performed using the Accelerated Solvent Extraction (ASE 350 Dionex, Thermo Fisher Scientific, Rovigo, Italy) with chloroform:methanol (2:1, *v*/*v*). Samples were placed in 22 mL extraction cells (2.0 g), and a weighted amount of C21:0 was added as a standard for lipid determination. The sample loading was completed by filling with Diatomee hydromatrix (Agilent Technologies, Santa Clara, CA, USA) and sealing off each extraction cell with a cellulose filter. After extraction, the solvent was evaporated with Univapo 100 ECH (Vacuum concentrator centrifuge, Elettrofor, scientific instrument, Rome, Italy) and then with a nitrogen stream until a constant weight was achieved. The extract weight (fat) was obtained by subtracting the vial tare from the total weight. The samples were finally stored in hexane in order to carry out derivatization. Fatty acid methyl esters (FAME) were prepared using methanolic HCl [31]. Lipid samples were mixed with 2 mL of hexane and 3 mL of methanolic HCl in 20 mL glass tubes with Teflon lined caps. The mixture was heated at 70 °C for 2 h and then cooled to room temperature. FAMEs were extracted in 2 mL of hexane after the addition of 5 mL of 6% (*w*/*v*) K_2_CO_3_ and Na_2_SO_4_ anhydrous. Samples stayed for 30 min prior to centrifugation at 1000 g for 10 min at 20 °C. The upper lipid layer was then removed, concentrated under N_2_ and diluted in hexane. FAMEs were analyzed using a GCMS 5977E MSD (Agilent Technologies, Santa Clara, CA, USA) and separated with a HP-88 capillary column (0.25 mm × 100 m and a film coating thickness of 0.25 µm; Supelco Inc., Bellafonte, PA, USA). The initial column temperature was 50 °C (hold for 5 min), ramped to 160 °C at 8 °C/min (hold for 4 min), ramped to 200 °C at 0.6 °C/min (hold for 1 min), and finally ramped to 240 °C at 15 °C/min (hold for 5 min). Helium was used as the carrier gas at the rate of 1.2 mL/min, and FAMEs were identified using NIRST Library and external standards (Supelco 37-component FAME mix including conjugated linoleic acids; Sigma-Aldrich, Milano, Italy). FAMEs were quantified using C21:0 as the internal standard and were expressed as the percentage of the total FA that were identified.

The atherogenicity index (AI) was calculated according to Ulbricht and Southgate [32].

AI = (C12:0 + 4 × C14:0 + C16:0)/(monounsaturated FA, MUFA + PUFA);

Desirable fatty acids (DFAs) were calculated as reported by Pilarczyk and Wójcik [33].

DFA = MUFA + PUFA + C18:0

Hypocholesterolemic/Hypercholesterolemic ratio (h/H) was calculated according to Chen and Liu [34].

h/H = (C18:1n-9c + C18:1n-11c + C18:1c + PUFA)/(C12:0 + C14:0 + C16:0);

∆9 desaturase index was calculated according to Bartoň et al. [35].

∆9 desaturase index = 100 × (C14:1n-9c + C16:1n-9c + C18:1n-9c + CLA)/(C14:1n-9c + C16:1n-9c + C18:1n-9c + CLA + C14:0 + C16:0 + C18:0 + C18:1t).

### 2.4. Statistical Analysis

Statistical analysis was performed using R software vers. 4.0.4 [36]. The normality of data distribution was tested using the Shapiro–Wilk test. In the present trial, treatment (HEMP and SB) was the factor of interest, whereas diet (H, CS and Pa) was considered as a nuisance factor. To control diet-related variability, data were analyzed using a linear model with the diet and treatment groups considered as block and fixed factors, respectively. Despite being similar between treatment groups and even within diet groups, the age of animals was initially included in the model as a covariate because of its variability (coefficient of variation, 39%). When the model assumptions were violated, a robust linear model was conducted (package robust) [37]. Moreover, when the effect of age was non-significant, it was removed from the model. Relationships between variables were assessed using Pearson’s or Spearman’s correlation coefficients, when appropriate. Data are presented in text as mean ± standard error. 

## 3. Results

Under H diet, hay was made of CP 71 g/kg DM, NEl 4.3 MJ/kg DM, and hay intake was 9.0 and 9.3 kg DM for SB and HEMP, respectively. Under CS diet, corn silage was made of CP 78 g/kg DM, NEl 5.9 MJ/kg DM, and corn silage intake was 13.1 and 12.2 kg DM for SB and HEMP, respectively. During grazing for the Pa diet, grass was made of CP 158 g/kg DM, NEl 5.8 MJ/kg DM, and grass intake was 7.9 and 7.1 kg DM for SB and HEMP, respectively (data not reported in Tables). In all dietary groups, the offered concentrate was completely consumed by cull dairy cows. Consequently, the average hempseed intake was 4.5% of the total DM intake both in H and CS and Pa. The diets were isocaloric both in H (5.9 and 5.7 MJ/kg DM intake for SB and HEMP, respectively) and CS (6.3 MJ/kg DM intake both for SB and HEMP) and Pa (6.6 MJ/kg DM intake both for SB and HEMP). Moreover, the diets were isonitrogenous on DM basis both in H (10.1 and 10.0% CP intake for SB and HEMP, respectively) and CS (10.9% CP intake both for SB and HEMP) and Pa (15.5% and 15.3% CP intake for SB and HEMP, respectively; data not reported in Tables). The average calculated chemical composition of diets of SB and HEMP is reported in Table 1.

Hempseed cake did not affect daily gain and efficiency of gain (gain/feed ratio; *p* > 0.05). The similar initial body weight and daily gain led to a similar body weight at slaughter (*p* > 0.05). Dressing percentage and carcass characteristics such as weight, conformation and fatness score were also unaffected (*p* > 0.05; Table 2) by the inclusion of hempseed in the diets of cull dairy cows. HEMP had pH 48 h and meat characteristics in terms of cooking loss, WBSF, composition and color, similarly to SB (*p* > 0.05; Table 3).

Despite the fact that the FA composition of the experimental meats was not influenced by the use of hempseed cake in the diets of cull dairy cows (*p* > 0.05; Table 4), SB had a higher DFA (*p* < 0.05) and a tendentially higher h/H ratio (*p* < 0.10) than HEMP. HEMP had a similar Δ9 desaturase index to SB (*p* > 0.05; Table 4).

Significant negative correlations were found between Δ9 desaturase and odd and branched-chain fatty acids (OBCFA; r = −0.625; *p* < 0.01), C17:0anteiso (r = −0.437; *p* = 0.03), C17:0iso (r = −0.723; *p* < 0.01), C18:3n-3 (r = −0.527; *p* < 0.01), while the correlation between Δ9 desaturase and PUFAn-3 was r = −0.379 (*p* = 0.06; data not reported in Tables).

## 4. Discussion

At slaughter, almost all animals had a rumen pH within the range considered normal, 6.2–7.2 [38], and all animals showed a rumen pH above 5.5 and 6.0 (Table 2), which are pathognomonic for acidosis and for the possible detection of early lactic acidosis, respectively [39]. As expected, considering the large use of forage (63% on average), the ruminal fermentations can be considered normal.

Hempseed cake did not affect daily gain and gain/feed ratios probably because the experimental diets were isocaloric and isonitrogenous. However, Semwogerere et al. [13] reported that hemp by-products are deficient in lysine, which is a growth-limiting amino acid, compared to soybean meal. In the present trial, no negative effect of hempseed cake on animal performance was observed, probably because of the modest daily gain or because lysine deficiency was counterbalanced by the high level of rumen undegradable proteins that characterize hemp by-products [40,41]. HEMP had similar dressing percentage and carcass characteristics to SB. In agreement with the present results, Gibb et al. [17] failed to highlight an effect of the inclusion of hemp seed in the diet of steers on daily gain, gain efficiency and dressing percentage, even at an inclusion level of 14%. Hessle et al. [18], feeding dairy steers with hempseed at about 10% of DM intake in replacement of soybean, found no effect on dressing percentage, carcass weight, conformation and fatness score. In general, the absence of effects on in vivo performance and carcass traits in the present study agrees with previous studies on fattening cattle aimed at substituting soybean meal with alternative protein sources in isonitrogenous and isocaloric diets [42,43]. However, Winders et al. [20], feeding heifers a diet containing 20% DM of hempseed cake, observed a reduction in average daily gain, but no effect on carcass characteristics was observed compared to the control group. Gallo et al. [44], considering IS cull dairy cows slaughtered during a 6-month period in the Autonomous Province of Trento (Italy), found the average slaughter weight, 667 kg; dressing percentage, 44.1%; average conformation scores, 1.58 points; and fatness scores, 1.7 points to be lower than those found in the present study. However, in the above cited study, it is not reported whether the animals were fattened before slaughter.

HEMP had similar pH 48 h and meat characteristics to SB. Most LT showed a pH 48 h within a normal range, 5.40–5.59, and none exceeded the value of 5.87, which can lead to dark cutting meat [45]. Meat characteristics, such as color, cooking loss and shear force, were evaluated after 14 days of aging, when aging is almost complete [46], and to our knowledge, only one study carried out on cattle is available. Turner et al. [19] observed that hempseed cake added at a level of about 10% DM to the diet of steers did not influence the shear force of the meat. The average WBSF was 37.4 ± 1.25 N. According to values provided by Miller et al. [47], the consumer acceptability rating for these meats would be over 93%. In addition, the meat of the animals in this study can be considered tender. In fact, according to Boleman et al. [48], meat can be classified as tender if it has values near or below 35 N, and it can be considered as hard if it has values above 58 N. The solubility of muscle collagen has a direct influence on the tenderness of the meat [49]. Since cull cows have a lower percentage of soluble collagen than younger cattle [49], tough meat might be expected. The low WBSF values observed in this study can be explained because, as reported by Alvarenga et al. [50], LT is a muscle with low connective tissue content and, consequently, WBSF in this muscle is mainly related to protein degradation. The level of intramuscular fat, 4.18 ± 0.382, may also contribute to explain the WBSF results. Indeed, this value was higher than that obtained by Corazzin et al. [51] in meat from younger animals: 3.2%. Dos Santos Fontes et al. [49] observed that intramuscular fat tended to be higher in cull cows than in heifers fed the same diet. In general, the average fat content of the meat found in this study is higher than the minimum value proposed by Savell and Cross [52], 3%, which is necessary to ensure the good acceptability of meat by the consumer. In the present study, the color of meat was not affected by the inclusion of hempseed cake in the animals’ diet. Antunović et al. [53] found no changes in the color of lamb meat when hempseed cake was added at the level of 12% as fed in the diet as a partial replacement for soybean. 

HEMP meat had similar FA compositions to SB meat. Gibb et al. [17] showed that including hempseed in the diet increased saturated and decreased unsaturated FA in *pars costalis diaphragmatic* fat, but not in brisket fat, and only at the higher level considered, 14% as fed, suggesting that the effect of hempseed is limited and muscle dependent. Turner et al. [19] showed that *longissimus dorsi* m. of steers supplemented with hempseed cake increased C18:1n-9c, C18:1n-11t and CLA and decreased C16:0 without affecting SFA and PUFA compared to the control group. Despite the similar content of PUFAn-3 and PUFAn-6, the above cited authors observed a reduction in meat from hempseed supplemented steers of the PUFAn-6/PUFAn-3 ratio, which was defined of small biological significance by the same authors. These results could be due to different levels of dietary PUFA or the different intensities of ruminal biohydrogenation between the experiment groups. Despite the high levels of PUFA in the diet, Turner et al. [54] showed that a 22% DM inclusion of hempseed cake in the lamb diet had only a minor effect on the PUFA content of the meat, with the exception of C22:6n-3, and no effect on the PUFAn-6/PUFAn-3 ratio probably due to extensive ruminal biohydrogenation. The results of the present study may be due to similar dietary FA intake and rumen biohydrogenation between HEMP and SB. In fact, the experimental groups had similar C18:3n-3 and C18:2n-6 that have only dietary origin and had similar intermediates, C18:1n-11t and C18:1t, and end product, C18:0, of biohydrogenation of dietary PUFA. OBCFA, which is an indicator of the amount of bacterial matter leaving the rumen as reported by Vlaeminck et al. [55], and iso and anteiso FA, which are related to the presence of cellulolytic and amylolytic bacteria respectively [56], were also similar between experimental groups. The Δ9 desaturase index was similar between HEMP and SB. ∆9 desaturase is an enzyme involved in the conversion of dietary or de novo synthesized saturated fatty acids (SFA) into MUFA [57]. Mierliţá et al. [58] observed increased Δ9 desaturase in the mammary gland and probable incomplete rumen biohydrogenation of dietary unsaturated FA with the inclusion of hemp in goat diets. In the present study, negative correlations were found between Δ9 desaturase index and FA or group of FA, such as OBCFA, C17:0anteiso, C17:0iso, C18:3n-3 and PUFAn-3. In general, Vlaeminck et al. [56] observed that branched C17 FA was negatively related to de novo synthesized FAs; similarly, Wongtangtinharn et al. [59] reported that branched FA can reduce FA synthesis. Waters et al. [60] reported a negative relationship between Δ9 desaturase gene expression and concentrations of PUFAn-3 and C18:3n-3 in beef.

The mean levels of PUFA, 4.01 ± 0.137%, and PUFAn-3, 0.55 ± 0.032%, were rather similar to those reported by Pilarczyk and Wójcik [33] in Simmental young bulls slaughtered at 25 months of age. Furthermore, Stelzleni and Johnson [61] showed a PUFA level of 4.78% in cull dairy cow meat, and it is interesting to note that the mean PUFAn-6/PUFAn-3 ratio observed in the present study, 6.40 ± 0.502, was not far from the maximum ideal value suggested by Wijendran and Hayes [62] for foods and reported by Pilarczyk and Wójcik [33], 6.0. The atherogenicity index, which quantifies the risk of cardiovascular disease related to the consumption of a food, should be less than 1 [33], in agreement with the average value of 0.93 ± 0.031 obtained in the experimental meats. Although both experimental groups showed high values of DFA and h/H ratio compared to those reported by Pilarczyk and Wójcik [33] for different cattle breeds, SB had a higher DFA and a tendentially higher h/H ratio than HEMP. From this point of view, SB meat was more beneficial to human health than HEMP meat.

## 5. Conclusions

Hempseed cake included in the diet of Italian Simmental cull dairy cows within the levels provided by EFSA [24], 5% of DM intake, can successfully replace soybean meal without affecting in vivo performance, carcass characteristics and meat quality in terms of color, cooking loss and shear force. The FA composition of intramuscular fat was similar between experimental groups, with the exception of the DFA level, which was lower in the HEMP group. The modest level of hempseed cake inclusion in the cull dairy cows diets suggested by EFSA [24] and/or the fact that the diets were isocaloric and isonitrogenous could contribute to explain these results.

## Figures and Tables

**Table 1:** Average chemical composition of diets.

	SB	HEMP
Chemical composition (unit kg^−1^ DM)		
Crude Protein (g)	120.5	119.2
Crude fiber (g)	191.5	197.5
Ether extract (g)	23.9	27.2
Ash (g)	73.2	71.7
NEl (MJ)	6.2	6.2
Fatty acid composition (g kg^−1^ fatty acids)		
C14:0	7.0	6.6
C16:0	228.3	215.9
C18:0	27.1	27.8
C18:1n-9	147.6	139.6
C18:2n-6	391.9	397.5
C18:3n-3	158.6	166.1

SB = Animals fed with soybean meal in concentrate; HEMP = Animals fed with hempseed cake in concentrate; DM = dry matter; NEL = Net energy for lactation, calculated according to INRA standards [23].

**Table 2:** Performance of Italian Simmental cull cows fed soybean meal (SB) and hempseed-supplemented (HEMP) diet, n = 26.

Item	Diet		RSD	*p*-Value
	SB	HEMP		
Slaughter weight (kg)	700.9	717.1	79.96	0.611
Daily gain (kg/d)	0.794	0.826	0.287	0.780
Carcass weight (kg)	348.3	344.2	43.16	0.812
Dressing Percentage (%)	49.7	48.1	2.587	0.138
Gain/feed ^1^	0.051	0.057	0.015	0.386
Intake DM (kg/d)				
Forage	9.95	9.52	2.18	0.621
Total	14.9	15.2	2.32	0.706
Carcass				
Conformation score (points) ^2^	2.62	2.39	0.599	0.337
Fatness score (points) ^3^	2.33	2.33	0.649	0.999
pH rumen	6.53	6.54	0.177	0.836

RSD = residual standard deviation; DM = dry matter. ^1^ Age of animals was include in the statistical model as coviarate. ^2^ S = 6 (superior), E = 5, U = 4, …, P = 1 (poor). ^3^ Class 5 = 5 (very fat), …, class 1 = 1 (very lean).

**Table 3:** Meat quality of Italian Simmental cull cows fed soybean meal (SB) and hempseed-supplemented (HEMP) diet, n = 26.

Item	Diet		RSD	*p*-Value
	SB	HEMP		
pH 48 h	5.45	5.48	0.029	0.910
Cooking Loss (%)	27.76	28.70	3.364	0.485
WBSF	37.57	37.27	6.397	0.907
Composition				
DM (%)	26.15	26.54	1.511	0.516
Ash (%)	1.05	1.03	0.034	0.409
Ether extract (%)	3.89	4.55	1.657	0.324
Protein (%)	20.34	20.23	0.502	0.586
Color				
L*	31.66	32.86	1.733	0.199
a*	16.17	15.91	1.930	0.741
b*	16.13	16.13	1.357	0.999

RSD = residual standard deviation; DM = dry matter; WBSF = Warner–Bratzler shear force; L* = lightness; a* = redness; b* = yellowness.

**Table 4:** Fatty acid (FA) composition (g/100 g of total FA) of *Longissimus thoracis* m. of Italian Simmental cull dairy cows fed soybean meal (SB) and hempseed-supplemented (HEMP) diet, n = 26 ^1^.

Item	Diet		RSD	*p* Value
	SB	HEMP		
C14:0	3.14	3.39	0.461	0.071
C15:0iso	0.21	0.22	0.043	0.952
C15:0anteiso	0.20	0.29	0.076	0.387
C14:1n-9c	0.28	0.24	0.143	0.493
C15:0	0.44	0.47	0.093	0.762
C16:0iso	0.23	0.26	0.074	0.238
C16:0	27.77	28.42	2.060	0.101
C17:0iso	0.32	0.34	0.061	0.378
C16:1n-7c	0.27	0.24	0.050	0.972
C16:1n-9c	2.55	2.77	0.711	0.428
C17:0anteiso	0.64	0.64	0.140	0.933
C17:0	1.16	1.18	0.210	0.891
C17:1n-9c	0.54	0.52	0.116	0.626
C18:0	20.05	20.26	3.300	0.872
C18:1n-11t	1.34	1.58	0.515	0.247
C18:1t ^2^	0.38	0.29	0.105	0.434
C18:1n-9c	34.13	32.42	2.724	0.125
C18:1n-11c	0.97	0.96	0.196	0.863
C18:1c ^3^	0.61	0.63	0.129	0.622
C18:2n-6t	0.47	0.47	0.109	0.963
C18:2n-6c	2.28	2.37	0.376	0.559
C19:1	0.11	0.12	0.034	0.818
C20:0	0.17	0.17	0.066	0.182
C18:3n-3	0.40	0.43	0.089	0.315
C20:3n-6	0.13	0.13	0.047	0.832
C20:4n-6	0.23	0.18	0.080	0.898
CLA ^4^	0.31	0.26	0.117	0.256
OBCFA ^5^	3.27	3.47	0.763	0.507
SFA ^6^	54.65	56.00	3.569	0.294
MUFA ^7^	41.31	39.93	4.171	0.317
PUFA ^8^	4.05	4.07	0.540	0.910
PUFAn-3	0.55	0.57	0.131	0.702
PUFAn-6	3.19	3.25	0.485	0.764
PUFAn-6/PUFAn-3	6.08	6.50	1.451	0.801
AI ^6^	0.90	0.97	0.104	0.131
DFA ^7^	65.40	64.27	2.248	0.049
h/H ^8^	1.30	1.20	0.130	0.062
∆9 desaturase index ^9^	41.60	39.89	3.476	0.222

RSD = residual standard deviation; CLA = conjugated linoleic acid; OBCFA = odd and branched FAs; SFA = saturated FAs; MUFA = monounsaturated FAs; PUFA = polyunsaturated FAs; AI = atherogenity index; DFA = desirable fatty acids; h/H = hypocholesterolemic/hypercholesterolemic ratio. ^1^ Fatty acids detected at <0.1 g/100 g of total FA are not reported. ^2^ C18:1t = sum of t6-, t8-, t9-, t10-, t11-, t12-, t13, t14-18:1. ^3^ C18:1c = sum of c11-, c12-, c13-, c14-, c15-18:1. ^4^ Mixture of isomers. ^5^ corresponds to C14:0iso + C15:0iso + C15:0anteiso + C16:0iso + C17:0iso + C17:0anteiso + C13:0 + C15:0 + C17:0. ^6^ corresponds to (C12:0 + 4 × C14:0 + C16:0)/(MUFA + PUFA). ^7^ corresponds to MUFA + PUFA + C18:0. ^8^ corresponds to (C18:1n-9c + C18:1n-11c + C18:1c + PUFA)/(C12:0 + C14:0 + C16:0). ^9^ corresponds to (C14:1n-9c+ C16:1n-9c + C18:1n-9c + CLA c9, t11)/(C14:1n-9c+ C16:1n-9c + C18:1n-9c + CLA c9, t11 + C18:1n-11t + C14:0 + C16:0 + C18:0) × 100.

## Data Availability

The data presented in this study are available upon request.
